# Why so little effort to study anti-oxidant therapy in burns?

**DOI:** 10.1186/s41038-016-0056-6

**Published:** 2016-08-10

**Authors:** Gordon L. Klein

**Affiliations:** Department of Orthopaedic Surgery and Rehabilitation, University of Texas Medical Branch and Shriners Burns Hospital, 301 University Boulevard, Galveston, TX 77555-0165 USA

**Keywords:** Anti-oxidant, Burn, Oxidative stress

## Abstract

Given that oxidative stress is an inherent response to burn injury, it is puzzling as to why investigation into anti-oxidant therapy as an adjunct to burn treatment has been limited. Both the inflammatory response and the stress response to burn injury involve oxidative stress, and there has been some limited success in studies using gamma tocopherol and selenium to improve certain consequences of burns. Much remains to be done to investigate the number, doses and combinations of anti-oxidants, their efficacy, and limitations in improving defined outcomes after burn injury.

The publication by Adjepong et al. [1] points out that to date the literature contains 11 papers devoted to the efficacy of anti-oxidant therapy in wound healing in burn-injured people. This is surprising given that the pathophysiology of oxidative stress is inherent in the pathophysiologic response to burn injury. A recent review [2] discussed why burn injury gives rise to non-specific adaptive responses that lead to oxidative stress as well as reports of tissue-specific effects of oxidative stress on the bone, muscle, and neural tissue. Yet despite this knowledge, the frequency and severity of oxidative stress following burns are not documented nor is it agreed upon what actually constitutes anti-oxidant therapy, the correct dosages of anti-oxidants, or the timing of such treatment. The pharmacokinetics of anti-oxidants have not been reported in burns, so we lack basic information about the proper use of these agents nor do we know whether a single anti-oxidant is optimal treatment or whether a combination of anti-oxidants is required.

What we can do is to partially explain why oxidative stress occurs following burns and to mention the effects of oxidative stress on individual organ systems, focusing on the bone, muscle, and nerve tissue. The inevitability of oxidative stress comes from the observations that both the inflammatory response [2] and the glucocorticoid/stress response [2] to burn injury cause oxidative stress. While the mechanism by which inflammation and endogenous glucocorticoid production result in oxidative stress is not specified at present, in both the bone and muscle, these responses stimulate an up-regulation of the forkhead box O (FOXO) transcription factors. In the bone, an up-regulation of FOXO leads to its uptake by the nucleus and its binding to β catenin [3]. This prevents β catenin from participating in the Wnt signaling pathway that is critical to osteoblast differentiation and results in a reduction in bone formation [3]. Additionally, the up-regulation of FOXO factors results in a reduction of hydrogen peroxide in the mitochondria of osteoclast precursors resulting in a reduction in osteoclastogenesis and decreased bone resorption [4]. These data would then explain the hypodynamic bone observed in burn patients by the second week post-burn [5]. In the muscle, oxidative stress is associated with an up-regulation of FOXO transcription factors 1 and 3 which stimulate production of ubiquitin ligases, especially atrogin-1 and muscle ring finger protein 1 (MUrf1). These ligases break down the muscle, resulting in net muscle loss [6]. Moreover, glutathione S-transferase is also down-regulated in the muscles of thermally injured rats [7]. In neural tissue, there is an interaction between redox and autophagic pathways, such that the anti-oxidant lanthionine ketamine ethyl ester (LKE) improves some features of neurodegenerative disease [8].

Furthermore, some micronutrients considered to be anti-oxidants, such as zinc and copper, are lost following burns, by means of excessive excretion not only in urine but also by leakage from the burn wound [9]. Copper and zinc are critical components of the anti-oxidant enzyme superoxide dismutase, and stimulating this enzyme to function normally should be one of the objectives of anti-oxidant therapy. In one study by Berger’s laboratory [10], selenium losses were not excessive but intake was poor, leading to deficiencies.

Recently, attempts to treat the inhalation injury accompanying burns with the anti-oxidant vitamin E (γ tocopherol) in a sheep model have shown that a nebulized form of this compound can reduce oxidative stress and collagen deposition in airways following inhalation injury [11, 12], but we are still awaiting the results of any clinical trials in humans. The use of selenium in burns by Berger et al. [10] and others [13] has resulted in improved anti-oxidant status and more rapid wound healing. Thus, the proper use of anti-oxidants such as zinc, copper, selenium, and vitamin E could constitute the basis for developing a pharmacopeia of drugs used to combat oxidative stress in burned patients.

## Conclusion

In summary, the area of anti-oxidant therapy to prevent or treat consequences of burn injury represents a virtually unexplored area that holds promise for effective adjunctive therapy if only we begin to study anti-oxidants in a systematic and collaborative manner.
